# LINC01370 suppresses hepatocellular carcinoma proliferation and metastasis by regulating the PI3K/AKT pathway

**DOI:** 10.1007/s12672-024-01193-9

**Published:** 2024-08-01

**Authors:** Fei Xiao, Zhuoyun Zhang, Luqian Li, Xiaojie He, Yufeng Chen

**Affiliations:** 1 Laboratory Department, Maoming People’s Hospital, No. 101 Weimin Road, Maoming, 525000 Guangdong China; 2 Cancer Department, Maoming People’s Hospital, Maoming, 525000 China; 3 Department of Endocrinology, Maoming People’s Hospital, Maoming, 525000 China

**Keywords:** Hepatocellular carcinoma, LINC01370, Proliferation, Metastasis, PI3K/AKT pathway

## Abstract

**Background:**

Hepatocellular carcinoma (HCC) poses a serious threat to human health worldwide. lncRNA dysregulation is frequently observed in various cancers, including HCC. However, the function of LINC01370 in HCC progression and its underlying mechanisms remain unclear.

**Methods:**

LINC01370 expression in HCC tissues with cells was analyzed by applying the GEO and GEPIA databases and qRT-PCR. CCK-8 and Transwell assays were used to assess HCC cell proliferation, migration, and invasion. The PI3K, AKT, with p-AKT protein expression were analyzed by western blotting.

**Results:**

Gene Expression Omnibus (GEO) and Gene Expression Profiling Interactive Analysis (GEPIA) showed that LINC01370 expression was significantly lower in HCC tissues than in normal tissues. LINC01370 overexpression markedly repressed HepG2 SMMC-7721 cells proliferation, migration, and invasion. To understand the downstream mechanism of LINC01370 regulation, we further analyzed the genes co-expressed with LINC01370 in GSE136247 and GSE132037 and then performed KEGG analysis. The PA pathway was found to be a downstream pathway regulated by LINC01370 in GSE136247 and GSE132037 via gene co-expression and KEGG analysis. Furthermore, PI3K and p-AKT protein levels decreased after LINC01370 overexpression. Importantly, rescue experiments showed that activation of the PI3K/AKT pathway disrupted the repressive effect of LINC01370 overexpression on the proliferation, migration, and invasion of HepG2 of SMMC-7721 cells.

**Conclusions:**

This study verified that LINC01370 suppresses HCC proliferation with metastasis by regulating the PI3K/AKT pathway.

**Supplementary Information:**

The online version contains supplementary material available at 10.1007/s12672-024-01193-9.

## Background

Hepatocellular carcinoma (HCC) is a threat to human health in the world [1]. Given the failure of prophase indications, most patients with HCC are usually diagnosed at a later stage [2]. Despite curative improvements in therapeutic strategies for HCC have been made, the five-year total survival rate is usually unsatisfactory owing to the high proportion of recurrence with metastasis [3, 4]. Thus, to ameliorate HCC the prognosis of patients with HCC, it is essential to develop new early diagnostic biomarkers and molecular-targeted therapeutic strategies by expounding the latent molecular mechanisms underlying HCC tumorigenesis.

Increasing evidence suggests that long noncoding RNAs (lncRNAs), comprising over 200 nucleotides, are closely connected to physiological and pathological cellular processes, such as cell differentiation, proliferation, and metastasis [5, 6]. Recent studies have shown that aberrantly expressed lncRNAs participate in various biological processes involved in hepatocarcinogenesis [7]. Fu et al. [8] demonstrated that the lncRNA DNAJC3-AS1 is upregulated in HCC and calculated the pitiable survival; lncRNA DNAJC3-AS1 accelerates HCC by absorbing nascent miR-27b. Zhou et al. [9]reported that lncRNA ID2-AS1 could restrain HCC metastasis via the HDAC8/ID2 pathway. LINC01370 is a novel lncRNA. However, little information is available regarding its role in cancer progression. Dysregulation of the PI3K/AKT pathway (PA pathway), a typical survival pathway, is increasingly associated with HCC carcinogenesis[10]. However, whether LINC01370 is involved in regulating the PA pathway in HCC remains unclear.

In this study, LINC01370 expression in HCC tissues and cell lines was analyzed. Next, the effects of LINC01370 on HCC cell proliferation, migration, and invasion were assessed. Finally, we investigated whether LINC01370 regulates HCC cell proliferation and metastasis via the PA pathway.

## Materials and methods

### Bioinformatical analyses

The GEPIA 2 database was used to assess LINC01370 expression in 369 HCC tumor tissues and 160 normal tissues [11]. Relationships between LINC01370 and tumor TNM staging, disease-free survival, and overall survival were analyzed using GEPIA 2. Additionally, genes co-expressed with LINC01370 in the combined analysis of TCGA-LIHC tumor, TCGA-LIHC normal, and GTEx-liver were analyzed using GEPIA 2, and the top 300 co-expressed genes were chosen. Gene Expression Omnibus (GEO) was used to analyze differentially expressed genes in HCC by searching for liver cancer or HCC. Then GSE132037 and GSE136247 were chosen for further analysis [12, 13]. GSE136247 contains 69 samples, including 39 HCC and 30 non-tumor liver (NTL) tissue samples. GSE132037 contains 49 samples, including 29 HCC samples and 20 NTL tissue samples (GSE132037 originally contained 52 samples, but three recurrent samples were not included in this study). For GSE136247 and GSE132037 data processing, the Limma Bioconductor package (implemented in Transcriptome Analysis Console 4.0.2) was used to analyze expression data based on linear models [14]. A probe set is considered expressed if DABG values fall below the DABG threshold in at least 50% of samples within the dataset. The default DABG value was set as 0.05. A threshold of 0.7 was used to flag less desirable results. Exploratory Grouping Analysis (EGA) was used to analyze the relationships among groups of samples. Next, the number of probe set signals per sample was reduced to three for viewing, either linearly using PCA or t-distributed Stochastic Neighbor Embedding (t-SNE). Finally, the clustering analysis applies a label to each sample, indicating the grouping. Clustering analysis mechanisms include k-means, DBSCAN, and affinity propagation clustering. For normalization, normalizes and summarizes the data. This option summarized the probes as a single signal for each probe set. This process converts CEL files into CHP files. The analysis of variance (ANOVA) method (eBayes) was used to analyze differentially expressed genes.

### Cell culture with transfection

Four HCC cell lines (Hep3B, HepG2, SMMC-7721, and Bel-7402) and a normal liver cell line (HL-7702) were obtained from the Shanghai Academy of Life Science (Shanghai, China). The cells were cultured in DMEM (Gibco, Gaithersburg, MD, USA) supplemented with 10% FBS (Gibco) and 1%penicillin and streptomycin (Sigma, St. Louis, MO, USA). Cells were placed at 37 °C containing 5% CO_2_. LINC01370 (NR_109936) was synthesized by GENEWIZ (Suzhou, China), including ApaI and HindIII enzyme cleavage sites, then inserted into pcDNA3.1 plasmids to construct the LINC01370 overexpression plasmids (ov-LINC01370). Empty pcDNA3.1 plasmids were acted as nagetive control (NC). Cell transfections were carried out by applying Lipofectamine 2000 Reagent (Invitrogen, Carlsbad, CA, USA) per the manufacturer’s instructions. For cell transfections, total 3 µg ov-LINC01370 or NC plasmid was diluted to 100 µl Opti MEM medium as solution A. Total 3 µl of Lipofectamine 2000 was dissolved in 50 µl Opti MEM medium as solution B, mixing solution B for 5 min. Then solution A and B was mixed for 20 min before adding to a 6 well cell culture plate. After incubation for 5 h, the transdected-cells were cultured in complete culture medium for further study.

### qRT-PCR

Total RNA was separated using 1 ml TRIzol reagent (Invitrogen). The RNA concentration was determined using a spectrophotometer (NanoDrop, Wilmington, DE, USA). Total RNA was converted to cDNA using ImProm-II™ Reverse Transcription System Kit (Promega, Madison, WI,USA) and carried out in an ABI amplification instrument (Applied Biosystems, Foster City, CA, USA). Total 20 μl reaction solution was configured according to the manual, then incubate the reaction at 30 ℃ for 10 min, at 42 ℃ for 60 min, and at 85 ℃ for 10 min. The obtained cDNA was diluted by 20 times. Then 20 μl qPCR reaction system including 5 μl diluted cDNA was configured according to the instructions of the SYBR Green qPCR SuperMix kit (Invitrogen). The reaction conditions were as follows: 50 ℃ for 2 min, 95 ℃ for 2 min; Then, 95 ℃ for 15 s, 60 ℃ for 32 s, 40 cycles. qPCR reaction was carried out in the ABI PRISM® 7500 Sequence Detection System (Invitrogen). The primer sequences were as follows: LINC01370 forward: 5′- TTTTTATGGCTAGCGGCTGA-3′, reverse: 5′-CACACTTAAAATTGTTATGG-3′; U6 forward: 5′-CTCGCTTCGGCAGCACA-3′, reverse: 5′-AACGCTTCACG AATTTGCGT-3′. Fold changes in the transcripts were computed applying the 2^−ΔΔCT^ method; U6 serves as the internal reference. All experiments were independently repeated three times.

### Cell proliferation assays

Cell proliferation was assessed using the CCK-8 assay. The 1 × 10^4^ cells were inoculated into 96 well plates with three replicate wells per group. The cells were then incubated for 0, 24, 48, or 72 h. Then, 10 µL of CCK-8 solution was appended to every well at every point of time, and after 2 h of placed at 37 °C. The OD at 450 nm of each well was measured at 450 nm using a multiscan MK3 microplate reader (Thermo Fisher Scientific, Waltham, MA, USA). In addition, the cell number was captured by light microscopy (DMI6000B, Leica, Heidelberg, Germany). All experiments were independently repeated three times.

### Migration with invasion assay

Transwell chambers (Corning Costar, Cambridge, MA, USA) were used to analyze cell migration and invasion. The chambers were pre-coated with (for invasion) or without (for migration) 40 μl BD Matrigel. 1 × 10^5^ cells were resuspend in 100 μl serum-free medium and added to the top chamber with or without Matrigel in a serum-free medium, and the lower chamber contained 600 μl complete culture medium include 10% FBS. After 48 h of being placed at 37 °C with 5% CO_2_, non-migrated or non-invaded cells in the toper chamber were wiped by applying cotton swabs and then, cells in transwell chambers were stereotyped with 4% paraformaldehyde for 15 min and washed once with PBS. Next, the bottom surface cells of the membrane were stained with crystal violet for 10 min and washed once with PBS. Five regions were randomly selected, and the cells were counted using a microscope (Leica). All experiments were independently repeated three times.

### Western blotting

Total protein samples harvested from 1 × 10^6^ cells were acquired by applying 300 μl RIPA lysis buffer added with phenylmethanesulfonyl fluoride (PMSF) (Beyotime, Shanghai, China). Equal amounts of 20 μg protein lysates were loaded onto 10% SDS-PAGE gels. After electrophoresis, a PVDF membrane (Millipore) was used to 300 mA constant current electrotransfer the proteins. Then membrane was washed three times with TBST and sealed at 25 °C for 1 h with 5% skim milk powder solution. Then membrane was washed three times with TBST andincubated with primary antibodies overnight at 4 °C. Primary antibodies included anti-phosphoinositide 3-kinase (PI3K) antibody (1:1000 dilution; ab302958, Abcam), anti-phosphorylated (p)-protein kinase B (p-AKT) antibody (1:1000 dilution; ab8805, Abcam), and anti-AKT antibody (1:1000 dilution; ab38449, Abcam). Additionally, an anti-GAPDH antibody (ab181602, Abcam) was used at a 1:10000 dilution. After washed three times with TBST, membranes were soaked with HRP-labeled antibody (1:5000, ab205718, Abcam) for 2 h at 25 °C and then washed washed three times with TBST. Finally, the proteins were quantified using enhanced chemiluminescence (Keygentec, Nanjing, China) and ChemiDoc™ XRS systems (Bio-Rad). All experiments were independently repeated three times.

### Statistical analysis

Statistical analyses were performed using IBM SPSS version 19.0. All data were exhibited as means ± standard deviation (SD). Divergence was assessed using a *t*-test with a one-way ANOVA. *P*-values < 0.05 were considered as statistically divergences.

## Results

### LINC01370 was downregulated in HCC

**Fig.1:**
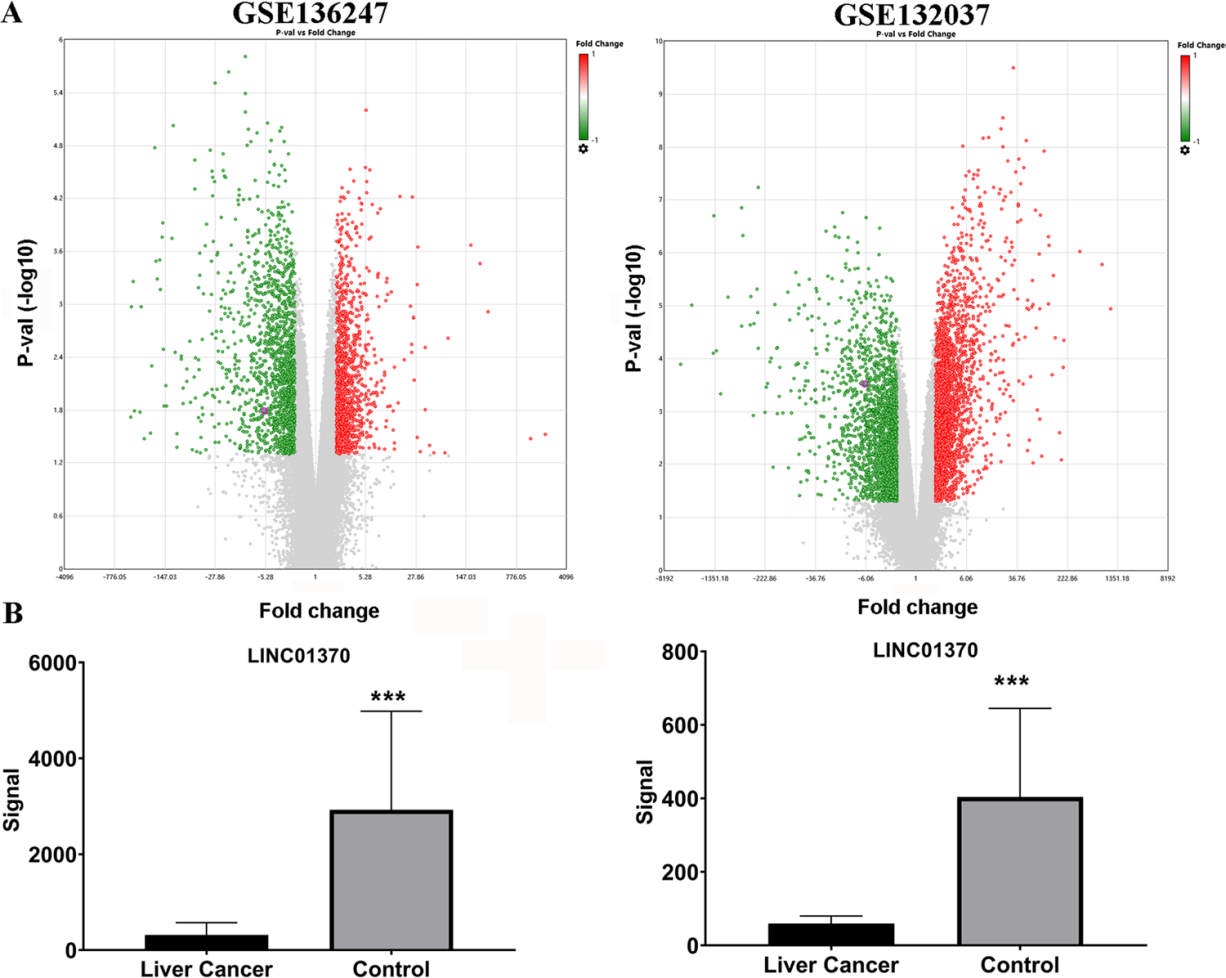
LINC01370 expression in HCC was analyzed via the GEO website. **A** DEGs between HCC tissues and control tissues in GSE136247 and GSE132037 were shown in the volcano plot. **B** LINC01370 expression was lower in HCC tissues in GSE136247 and GSE132037. GSE132037data were not included three recurrent samples in this study

**Fig. 2:**
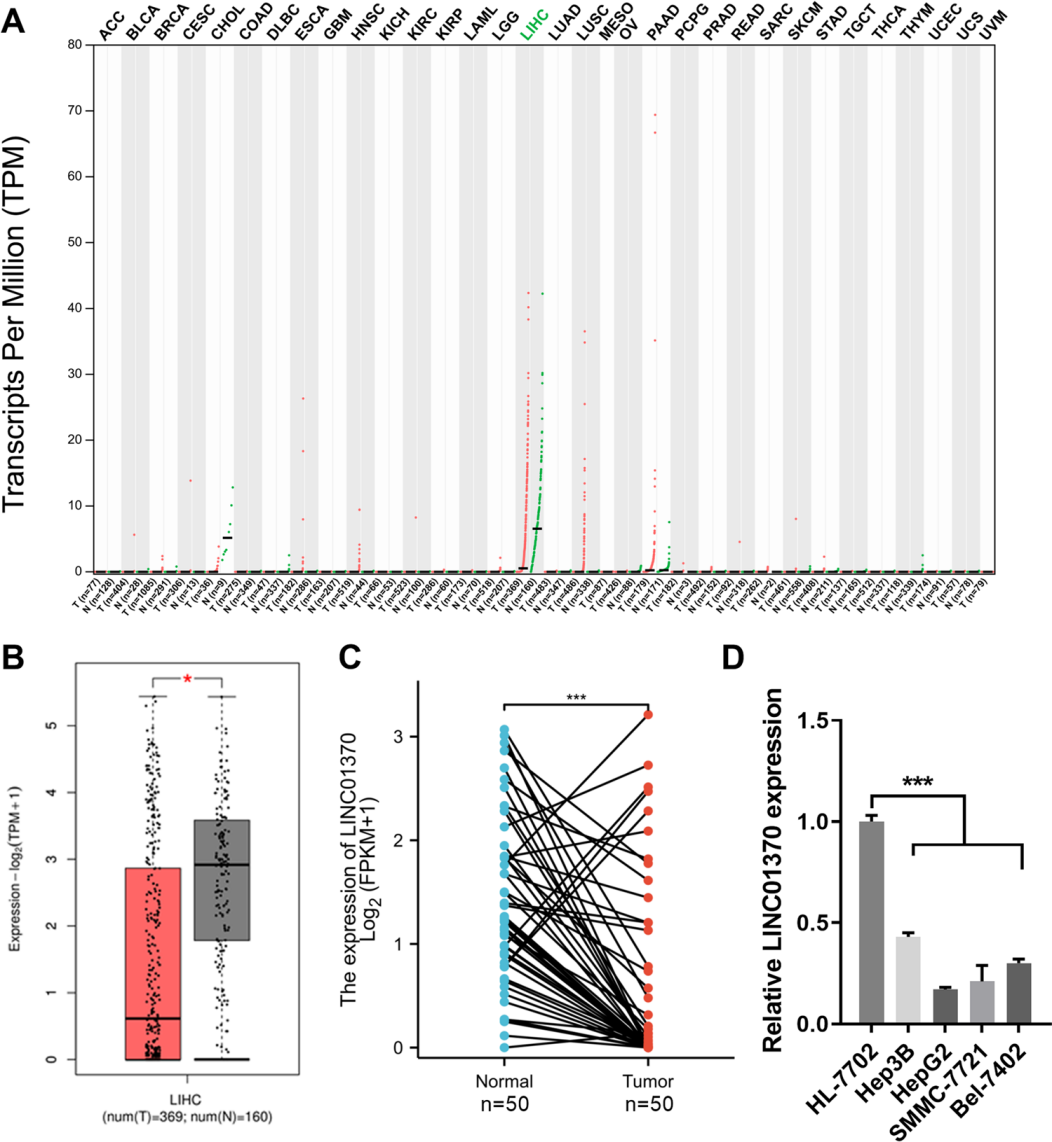
LINC01370 was reduced in HCC. **A** The LINC01370 expression in cancer tissues was assessed using the GEPIA online database. Green indicates low LINC01370 expression in tumors. **B** The LINC01370 expression in HCC (LIHC, Liver hepatocellular carcinoma) tissues was assessed using the GEPIA online database. **C** Differential expression of LINC01370 in HCC and corresponding normal liver tissue were analyzed through paired analysis. **D** qRT-PCR was used to evaluate the LINC01370 expression of HCC cells. (***P < 0.001)

To study LINC01370’s role in HCC, LINC01370 expression levels in HCC were first analyzed. Analyses of the GEO datasets GSE132037 and GSE136247 revealed that LINC01370 expression was significantly lower in HCC tissues than in normal tissues (Fig. 1). The GEPIA online database showed that LINC01370 expression was markedly downregulated in HCC tissues (LIHC) compared to that in normal tissues (Fig. 2A and B). Next, LINC01370 expression in HCC liver tissues was markedly downregulated compared with that in the corresponding normal liver tissues (Fig. 2C). However, LINC01370 expression did not significantly correlate with tumor TNM stage, disease-free survival, or overall survival in patients with HCC (Supplemental Fig. 1). LINC01370 expression in HCC and normal liver cell lines was assessed using qRT-PCR. The results revealed that LINC01370 expression in both HCC cell lines was dramatically decreased, especially in HepG2 and SMMC-7721 cells, compared to that in HL-7702 cells (Fig. 2D). Therefore, the HepG2 and SMMC-7721 cell lines were chosen for further study based on their low LINC01370 expression.

### LINC01370 overexpression suppressed cell proliferation, migration, and invasion

**Fig. 3:**
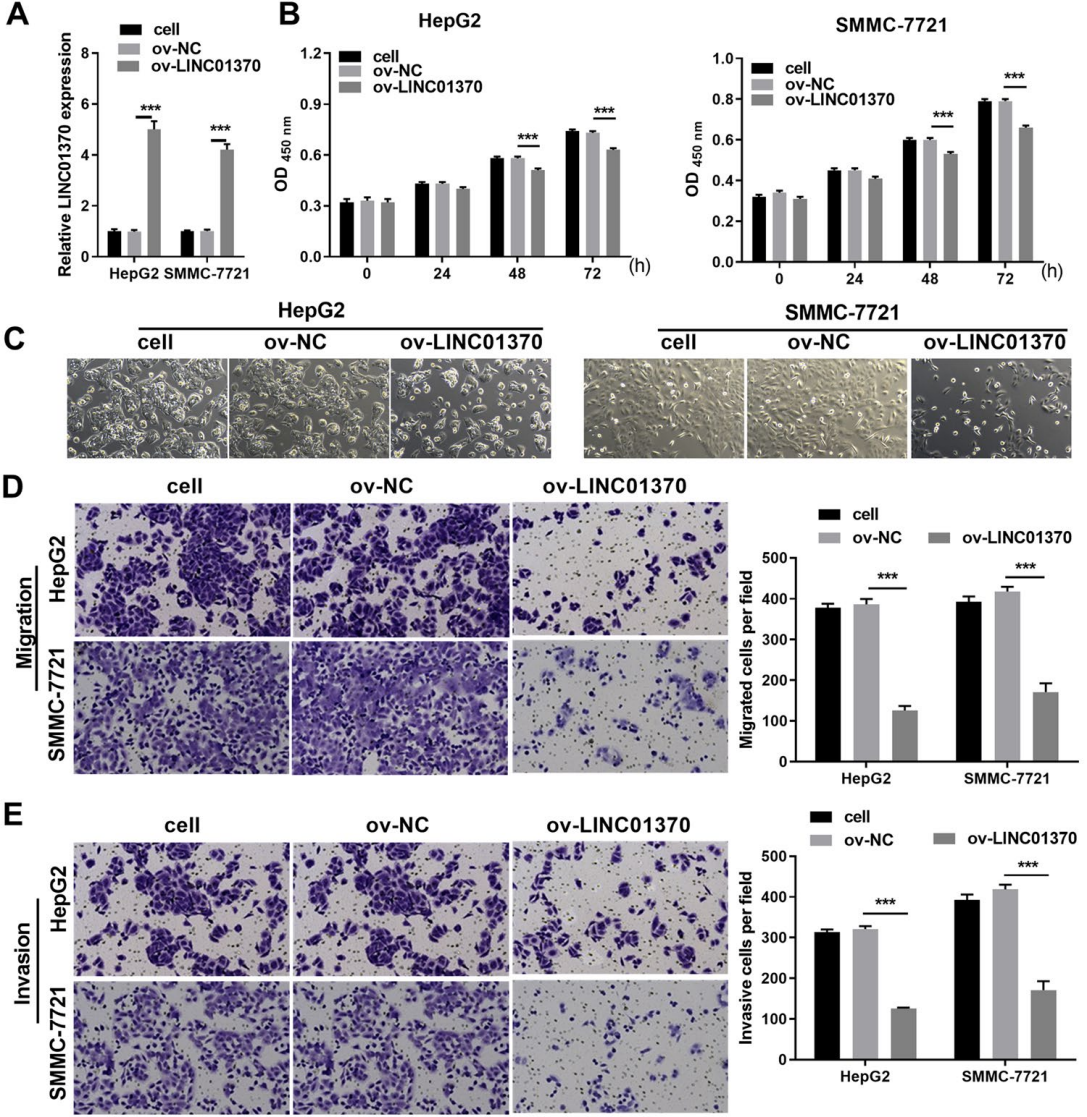
Function of LINC01370 overexpression in HCC cells. **A** The LINC01370 expression in two types of HCC cells transforming infection ov-LINC01370 or ov-NC was measured by applying qRT-PCR. **B** Cell proliferation of two types of HCC cells transfected with ov-LINC01370 or ov-NC was detected by CCK-8 assay. **C** Cell number of two types of HCC cells transfected with ov-LINC01370 or ov-NC was captured by light microscopy. **D** and **E** The migratory with invasive abilities of SMMC7721 with HepG2 cells transforming infection ov-LINC01370 or ov-NC were assessed applying transwell assay. (***P < 0.001)

To explore the functional effects of LINC01370 in HCC cells, HepG2 and SMMC-7721 cells were transformed with LINC01370 overexpression or negative control (NC) plasmids. qRT-PCR analysis showed that LINC01370 expression was significantly increased in both types of HCC cells transfected with the LINC01370 overexpression plasmid compared to that in NC-transfected cells (Fig. 3A). Functionally, the cell proliferation assay results indicated that LINC01370 overexpression remarkably suppressed the proliferation of the two types of HCC cells at 48 and 72 h compared to that in NC-transfected cells (Fig. 3B). Consistently, cell number was remarkably reduced in the LINC01370 group (Fig. 3C). Moreover, the transwell assay demonstrated that LINC01370 overexpression remarkably suppressed the migration and invasion abilities of the two types of HCC cells compared to those of NC-transfected cells (Fig. 3D and E).

### LINC01370 overexpression suppressed the PA pathway of HCC cells

**Fig. 4:**
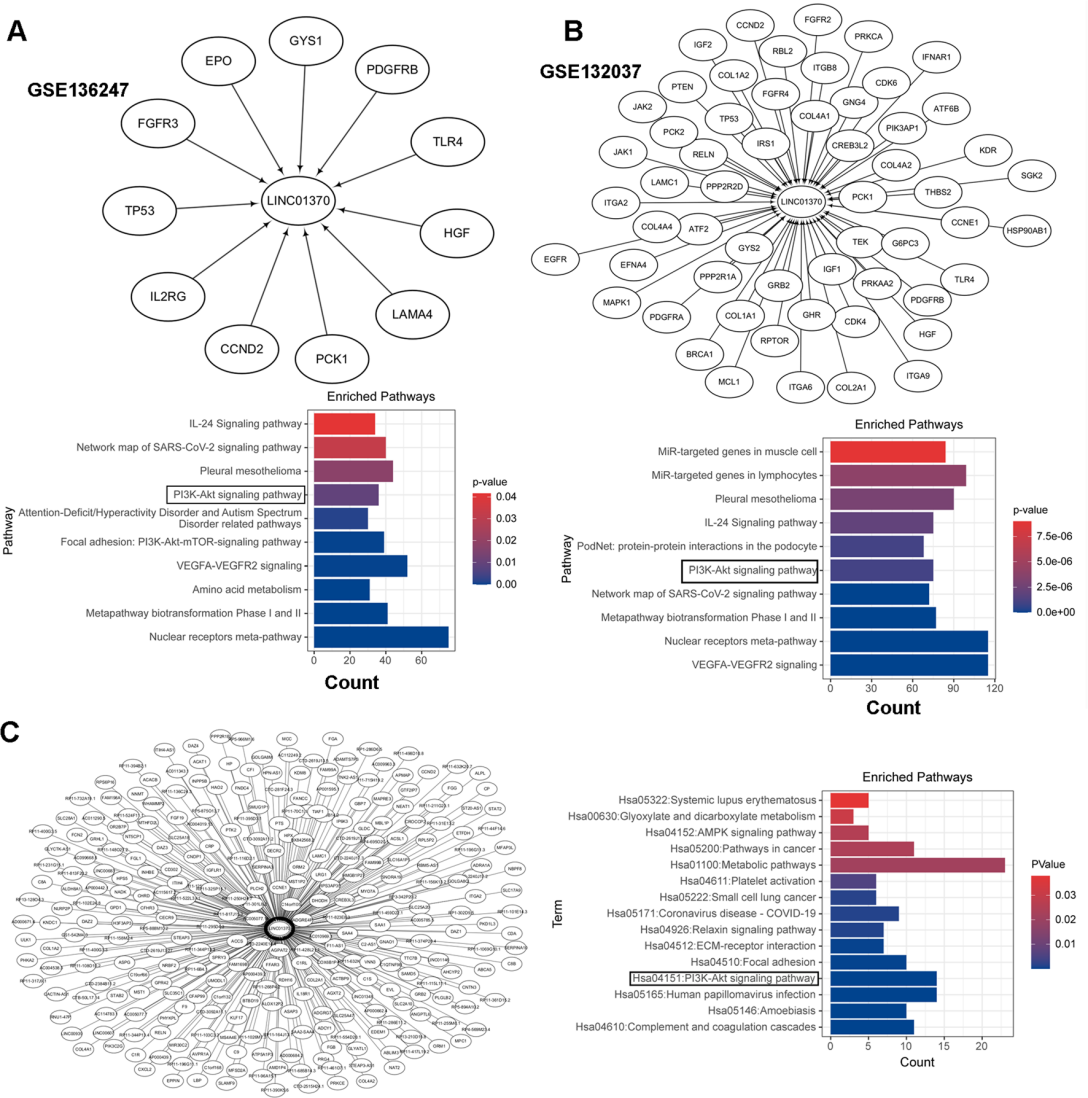
PA pathway was a potential downstream pathway regulated by LINC01370 in HCC cells. **A** and **B** The co-expressed genes of LINC01370 were analyzed in GSE136247 **A** and GSE132037 **B** and then performed KEGG analysis on the co-expressed genes. **C** The co-expressed genes of LINC01370 were analyzed in the combined analysis of TCGA-LIHC tumor, TCGA-LIHC normal, and GTEx-liver, and then performed KEGG analysis on the co-expressed genes

**Fig. 5:**
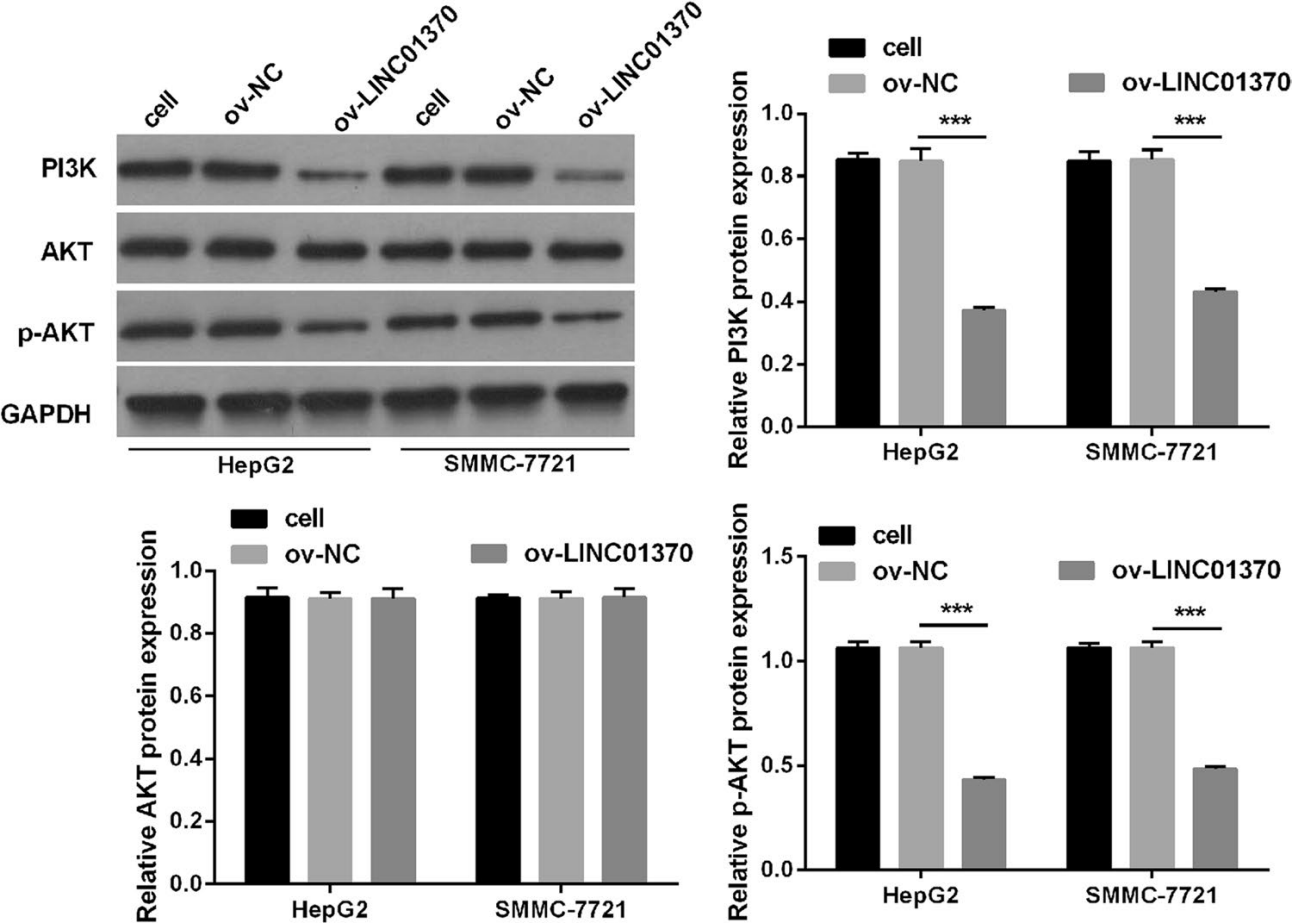
PA pathway was silenced by LINC01370 overexpression in HCC cells. The PI3K, AKT, and p-AKT protein expression in two types of HCC cells after LINC01370 overexpression were assessed by western blot. Original full-length blots were presented in Supplementary Fig. 2A. (***P < 0.001)

To understand the downstream mechanism of LINC01370 regulation, we further analyzed the genes co-expressed with LINC01370 in GSE136247 and GSE132037 and then performed KEGG analysis on the co-expressed genes. The PA pathway was found to be the downstream pathway regulated by LINC01370 in the GSE136247 and GSE132037 datasets (Fig. 4A and B). Additionally, the PA pathway was found to be a downstream pathway regulated by LINC01370 in the combined analysis of TCGA-LIHC tumors, TCGA-LIHC normal tissues, and GTEx-liver (Fig. 4C). The PA pathway has been verified as a vital HCC facilitation pathway [15]. Thus, we investigated whether the PA pathway was regulated by LINC01370. As expected, western blotting showed that LINC01370 overexpression markedly decreased PI3K and p-AKT expression in both types of HCC cells compared to NC cells (Fig. 5 and Supplemental Fig. 2A).

### Activating the PA pathway could reverse the repressive effect of LINC01370 overexpression on HCC cells

**Fig. 6:**
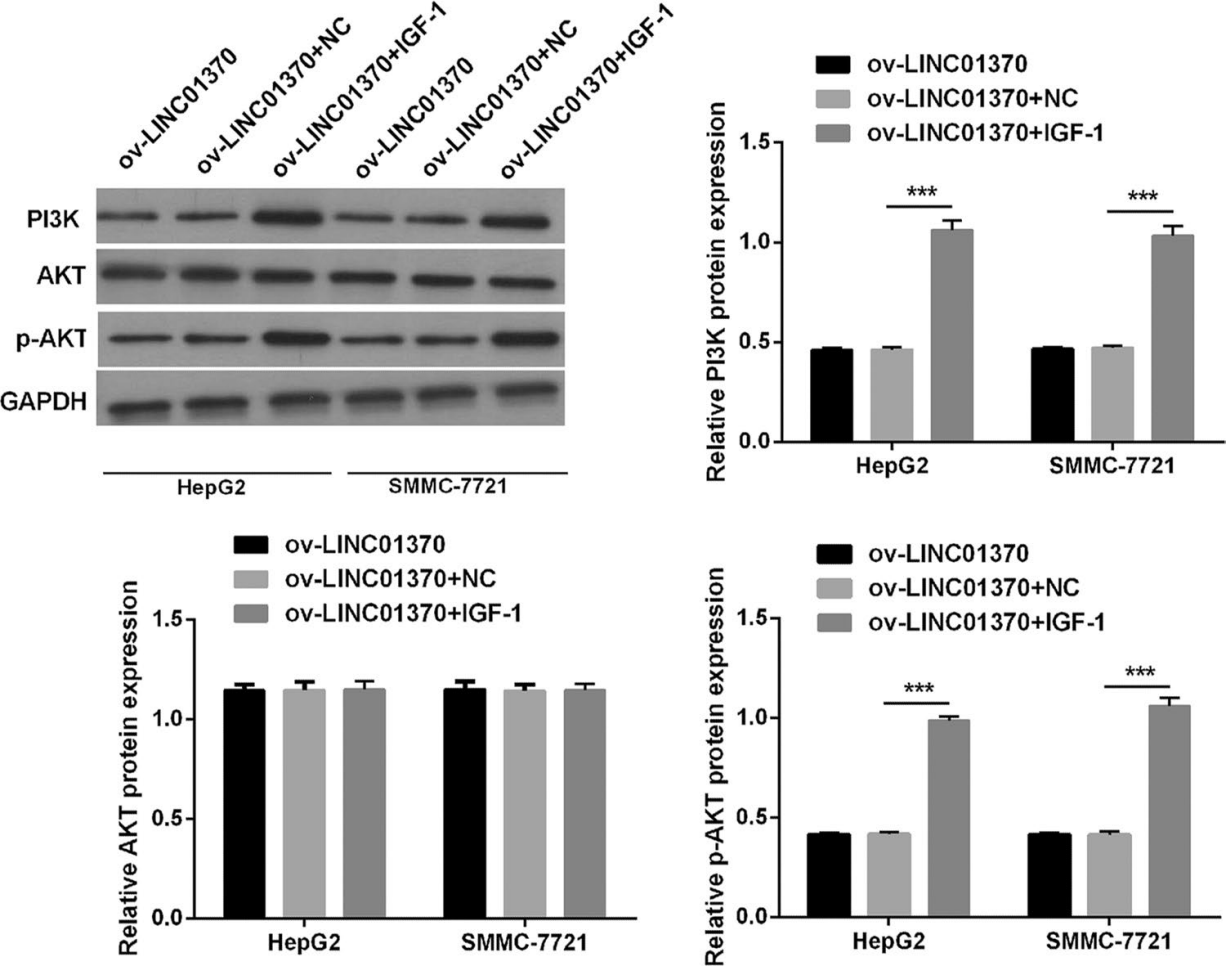
PI3K, AKT, and p-AKT protein expression in two types of HCC cells with LINC01370 overexpression c treated with IGF-1 were assessed applying western blot. Original full-length blots were presented in Supplementary Fig. 2B. (***P < 0.001)

**Fig. 7:**
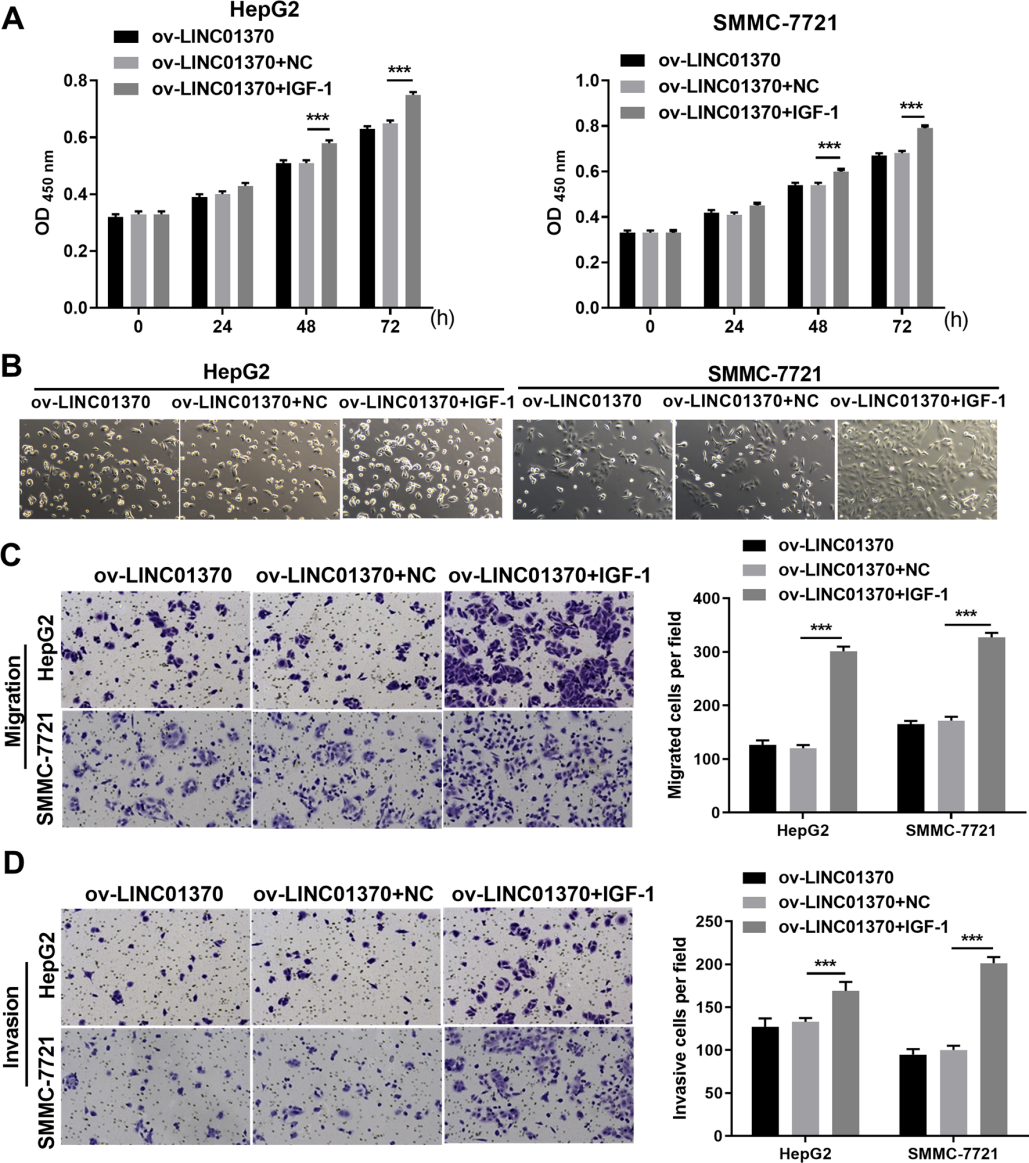
Activating PI3K/AKT pathway promoted proliferation, migration, and invasion of two types of HCC cells accompanying LINC01370 overexpression. **A** The proliferation of two types of HCC cells with LINC01370 overexpression after being treated with IGF-1 was analyzed by applying CCK-8 assay. **B** Cell number of two types of HCC cells with LINC01370 overexpression after being treated with IGF-1 was captured by light microscopy. **C** and **D** the migratory and invasive abilities of two types of HCC cells accompanying LINC01370 overexpression were analyzed by transwell assay. (***P < 0.001)

To confirm the role of the PA pathway downstream of LINC01370, HCC cells overexpressing LINC01370 were treated with a PI3K/AKT activator (IGF-1, 100 ng/ml; Sigma). PI3K and p-AKT protein expression was markedly increased in both SMMC7721 and HepG2 cells overexpressing LINC01370 (Fig. 6 and Supplemental Fig. 2B). We further investigated whether activating the PA pathway in the two types of HCC cells overexpressing LINC01370 could reverse the effect of LINC01370 overexpression in two types of HCC cells. Activating the PA pathway remarkably enhanced the proliferation, cell number, migration, and invasion abilities of the two types of HCC cells accompanied by LINC01370 overexpression (Fig. 7).

## Discussion

HCC is a medical condition with high morbidity worldwide, particularly in East Asia, South Asia, Africa, and Southern Europe [16]. Although numerous therapeutic approaches have been developed in recent years, the survival rate of HCC patients remains unsatisfactory [17]. Therefore, it is imperative to explain the theories of HCC progression by identifying novel diagnostic biomarkers and treatment strategies. Here, we confirmed that LINC01370 was abnormally downregulated in HCC tissues with HCC cells, LINC01370 was found to be a tumor inhibitory factor within HCC, and LINC01370 overexpression remarkably repressed HCC cell proliferation, migration, and invasion. Moreover, the PA pathway in HCC cells was clearly inhibited after LINC01370 overexpression. PA pathway activation could reverse the repressive effect of LINC01370 overexpression on the proliferation, migration, and invasion of HCC cells.

In recent years, the rapid expansion of molecular biology has accelerated the exploration of tumor development at the molecular level [18]. Numerous studies have reported that multiple lncRNAs are frequently aberrantly expressed in cancers and play vital roles in tumorigenesis, proliferation, metastasis, prognosis, and diagnosis [19–21]. HCC-related lncRNAs also have been shown to display aberrant expression and play a role in transformation processes, such as angiogenesis and epithelial-mesenchymal transition [22]. For example, LINC00578, RP11-298O21.2, RP11-383H13.1, and RP11-440G9.1 have been identified as being strongly associated with the prognosis of HCC, accurately predicting the 5-year survival rate [23]. lncRNA B3GALT5-AS1 expression was reduced within HCC, lncRNA B3GALT5-AS1 poses as a tumor inhibitory factor that inhibits the malignant features of HCC [24]. The lncRNA SNHG7 has been reported to accelerate cell growth and metastasis through miR-122-5p/FOXK2 in HCC [25]. LncRNA PP7080 can accelerate the proliferation, migration, and invasion of HCC cells through the miR-601/SIRT1 axis [26]. LncRNA DHRS4-AS1 inhibits HCC cell proliferation and colony formation through the miR-522-3p/SOCS5 axis [27]. LncRNAs can also exert their functions by directly binding to mRNA or proteins. LINC00707 binds to YTH N6-methyladenosine RNA-binding protein 2 (YTHDF2), leading to its ubiquitination-dependent degradation of the YTHDF2 protein, which inhibits natural killer cell antitumor activity in HCC [28]. The lncRNA PWRN1 maintains the high activity of pyruvate kinase M2 (PKM2) by binding to PKM2, which inhibits glycolysis and HCC growth [29]. SNHG9 inhibits EZH2 recruitment and reduces H3K27me3 levels in the PTEN promoter, thereby promoting EZH2 expression and inhibiting HCC development [30]. Finally, lncRNAs regulate the tumor microenvironment, leading to tumor immune tolerance [31, 32]. Therefore, it is important to observe and study the behavior of lncRNAs in patients with HCC. Here, we investigated the effect of LINC01370 on HCC cells and demonstrated that LINC01370 overexpression inhibited the proliferation, migration, and invasion of HCC cells.

The PA pathway has been reported to execute crucial actions in cell activities, including growth, proliferation, migration, and survival [33], and it is the most universally researched pathway in HCC, being activated in 40–50% of patients with HCC [34]. Recent studies have shown that lncRNAs can modulate cell proliferation, migration, and invasion by regulating the PA pathway in HCC [35]. Similar to the findings of Zhou et al., the lncRNA NCK1-AS1 aggravates the proliferation and migration of HCC cells by activating the PA pathway. Lai et al. [36] reported that LINC01572 promotes the proliferation, migration, invasion, and EMT of HCC cells by activating the PA pathway. In this study, we demonstrated that LINC01370 overexpression blocked PI3K and p-AKT protein expression. Moreover, rescue experiments confirmed that activating the PA pathway disrupted the repressive effect of LINC01370 overexpression on the proliferation, migration, and invasion of HCC cells, suggesting that LINC01370 suppressed the proliferation, migration, and invasion of HCC cells via regulation of the PA pathway.

Nevertheless, this study had some limitations. Although we assessed the effects of LINC01370 on the proliferation, migration, and invasion of HCC cells, we did not investigate the association between LINC01370 and xenograft carcinogenesis. Second, although we found that LINC01370 overexpression drastically inhibited the proliferation and metastasis of HCC cells by regulating the PA pathway, we did not make further efforts to probe its mechanism. In addition, the mechanism of action of LINC01370 needs to be verified through future in vivo experiments. Despite these limitations, our study emphasizes the possible role of LINC01370 as a novel therapeutic target for HCC.

In conclusion, our study demonstrated that LINC01370 expression was drastically reduced in HCC cells and LINC01370 overexpression suppressed HCC proliferation and metastasis by silencing the PA pathway. Thus, LINC01370 may be a promising therapeutic target for HCC.

### Supplementary Information

Additional file 1.

## Data Availability

All data generated or analysed during this study are included in this published article [and its supplementary information files].

## Abbreviations

HCC: Hepatocellular carcinoma
lncRNAs: Long noncoding RNAs
GEO: Gene expression omnibus
LIHC: Liver hepatocellular carcinoma
PA pathway: PI3K/AKT pathway
