# Supplementary material for: LINC01370 suppresses hepatocellular carcinoma proliferation and metastasis by regulating the PI3K/AKT pathway
Source: Discov Oncol. 2024 Aug 1;15:326. doi: 10.1007/s12672-024-01193-9 (PMC11294307; doi:10.1007/s12672-024-01193-9)
Supplement: Supplementary file 1 — Additional file 1. [file 12672_2024_1193_MOESM1_ESM.pdf]

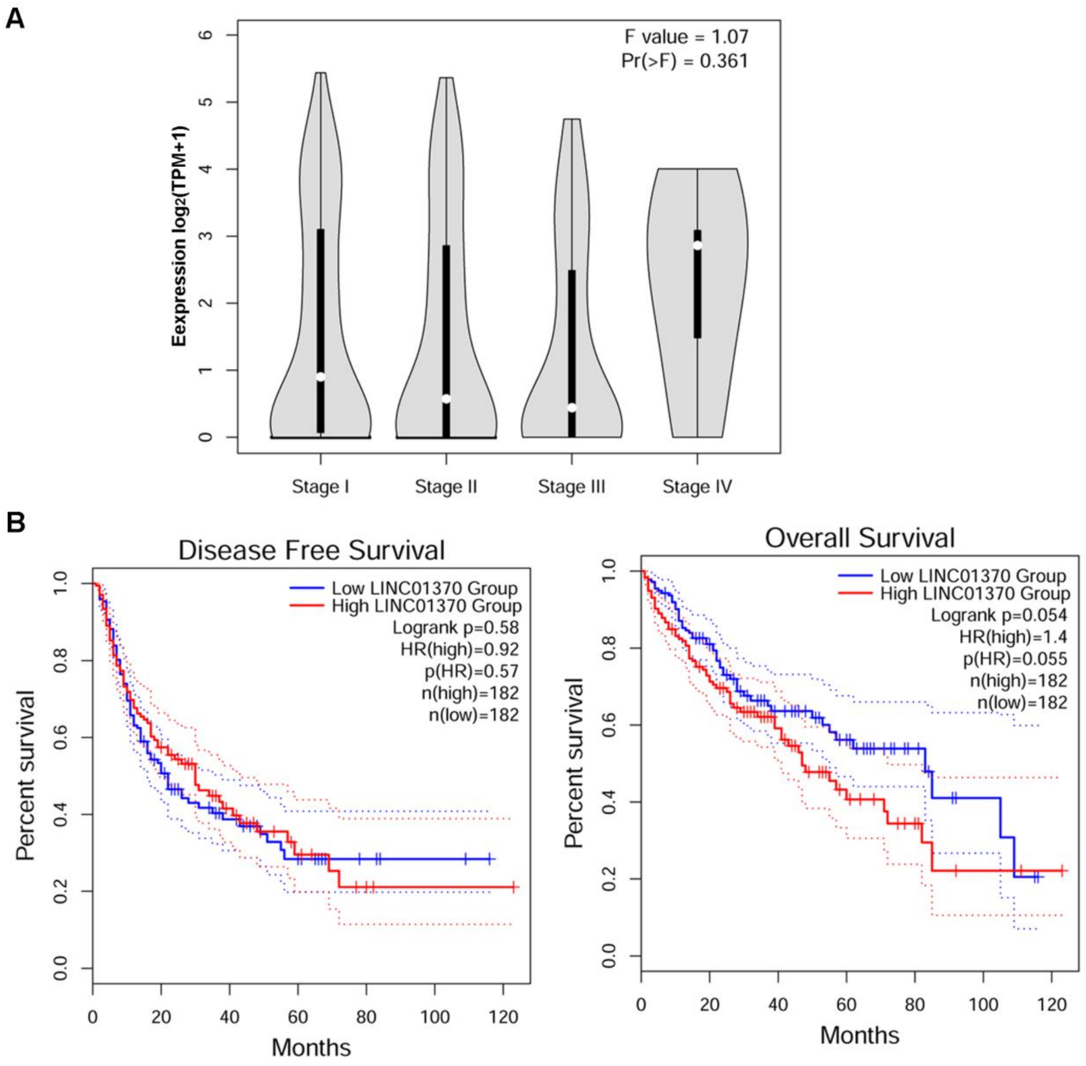

Supplemental Fig. 1 The relationship between LINC01370 and clinical significance was analyzed by GEPIA in patients with HCC. (A) The relationship between LINC01370 and TNM stage was analyzed by GEPIA. (B) The relationship between LINC01370, disease-free survival and overall survival was analyzed by GEPIA.

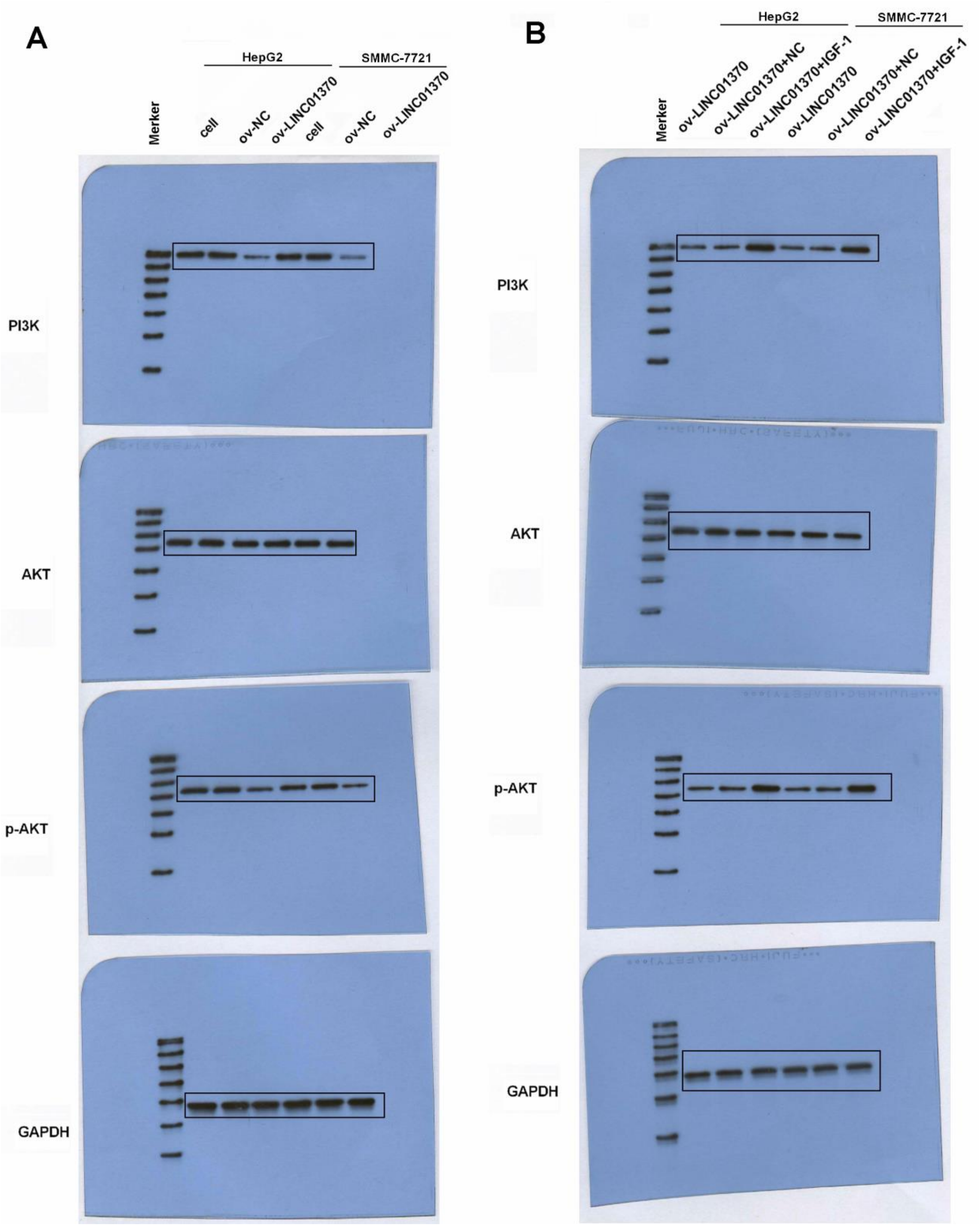

Supplemental Fig. 2 Full-length blots are presented from western blotting. (A) Full-length blots of Figure 5. The stripes inside the box are displayed in the Figure 5. (B) Full-length blots of Figure 6. The stripes inside the box are displayed in the Figure 6.
